# Age-Related Decline of Speech Perception

**DOI:** 10.3389/fnagi.2022.891202

**Published:** 2022-06-22

**Authors:** Ulrich Hoppe, Thomas Hocke, Heinrich Iro

**Affiliations:** ^1^Department of Audiology, ENT-Clinic, University of Erlangen-Nürnberg, Erlangen, Germany; ^2^Cochlear Deutschland GmbH & Co. KG, Hanover, Germany

**Keywords:** hearing loss, speech perception, age-related hearing loss (ARHL), random forest regression, machine learning, maximum word recognition, speech audiometry

## Abstract

Hearing loss is one of the most common disorders worldwide. It affects communicative abilities in all age groups. However, it is well known that elderly people suffer more frequently from hearing loss. Two different model approaches were employed: A generalised linear model and a random forest regression model were used to quantify the relationship between pure-tone hearing loss, age, and speech perception. Both models were applied to a large clinical data set of 19,801 ears, covering all degrees of hearing loss. They allow the estimation of age-related decline in speech recognition for different types of audiograms. Our results show that speech scores depend on the specific type of hearing loss and life decade. We found age effects for all degrees of hearing loss. A deterioration in speech recognition of up to 25 percentage points across the whole life span was observed for constant pure-tone thresholds. The largest decrease was 10 percentage points per life decade. This age-related decline in speech recognition cannot be explained by elevated hearing thresholds as measured by pure-tone audiometry.

## Introduction

More than 5% of the world’s population, approximately 460 million people, suffer from disabling hearing loss (WHO, 2021). Hearing disability is associated with reduced speech perception and, in consequence, reduced communication function. Hearing deteriorates with age (Zwaardemaker, 1891; Technical Committee ISO/TC 43 Acoustics, 2017). The ISO standard describes the age-dependent frequency-specific loss (ISO 7029:2017, 2017). The slope of the decline increases with growing age and frequency: While for 250 Hz the decline is in the order of 1 dB per decade in the fourth life decade, about 20 dB per decade can be observed for 6,000 Hz in the eighth life decade. While the ISO standard provides detailed information about the relationship between age and pure-tone sensitivity loss (PTSL), it makes no reference to speech recognition.

Given our ageing society and the prevalence of age-related hearing loss (ARHL), it is clear that hearing loss is a common public-health issue of increasing importance in the near future (WHO, 2021). Individuals with ARHL experience social withdrawal (Pronk et al., 2011), mental and physical decline (Shukla et al., 2020), and poorer quality of life (Davis et al., 2007).

Speech perception deficits in hearing-impaired people are mainly attributable to decreased audibility of the speech signal over part or all of the speech frequency range. Within Carhart’s (1951) framework for word recognition in quiet, this was referred to as *loss of acuity*. Additionally, Carhart introduced a second component which stems from impaired processing of the audible speech signal, resulting in a *loss of clarity*. Plomp (1978) referred to these components of hearing loss as *attenuation* (class A) and *distortion* (class D), respectively. The attenuation component can be assessed by pure-tone audiometry. The distortion component describes the impact of reduced temporal and frequency resolution. It is thought that the distortion component explains the deterioration of speech recognition which is not described by attenuation, namely pure-tone thresholds. Both attenuation and distortion are part of ARHL (van Rooij et al., 1989).

A large number of studies have focussed upon hearing in the elderly and have investigated PTSL and speech perception. However, the interpretation of these results remains challenging, as pure-tone thresholds change substantially with increasing age. Hence, it is necessary to correct for the effect of PTSL when investigating the effect of age on speech perception. One of the first attempts to do this was described by Jerger (1973) in a report on speech recognition in a large group of older subjects. He analysed scores from the clinical records of 2,162 patients. With subjects grouped according to age and average hearing loss at 0.5, 1 and 2 kHz, results suggested that speech recognition, defined as the maximum score (WRS_max_) obtained by using a monosyllabic word list, declines above the age of sixty. In particular, he found that age had an effect on speech recognition of approximately 4% per life decade for individuals with mild hearing loss, but that it had a greater effect (e.g., 10% per decade) upon those with higher degrees of hearing loss. Unfortunately, he did not report on hearing loss at higher frequencies. It is known for a long time that hearing thresholds at these higher frequencies are, in particular, worse for older subjects (Zwaardemaker, 1891; Technical Committee ISO/TC 43 Acoustics, 2017).

Several studies have revealed that deterioration in speech understanding occurs in addition to deterioration in hearing sensitivity and includes components beyond elevated hearing thresholds (Bergman et al., 1976; Jerger and Hayes, 1977; Marshall and Bacon, 1981; Pedersen et al., 1991; Divenyi and Haupt, 1997; Kronlachner et al., 2018).

Some authors (Dubno et al., 1997; Humes, 2007) have highlighted the challenge of separating varying auditory thresholds from age, a factor affecting all sensory modalities (Humes and Young, 2016). In recent studies, speech recognition and its relation to age were investigated either by correcting for PTSL (Hoppe et al., 2014; Müller et al., 2016) or by using a longitudinal study design (Dubno et al., 2008). In a clinical population Hoppe et al. (2014) investigated speech recognition with hearing aids and WRS_max_ for different age groups in relation to average hearing loss at 0.5, 1, 2, and 4 kHz (4FPTA). They found a monotonic decrease in speech recognition with increasing age and a significant drop of about 2–4% per decade. This drop was attributed to age-dependent distortion. Müller et al. (2016) investigated, as well, the WRS_max_ as a function of age. After correcting for 4FPTA they found a significant, though smaller, drop for people aged above 70 years of about 2–3% per decade. Neither study included a hearing threshold beyond 4 kHz, and therefore, a small overestimation of the influence of age cannot be excluded. However, Dubno et al. (2008) found a larger effect, around 7–8% per life decade. They performed a longitudinal study including 256 subjects with age-related hearing loss, aged 50–82 years, over a period of 3–15 years. The speech recognition scores were corrected for by changing hearing thresholds during the observation phase; this was done by using the individuals’ articulation index as an importance-weighted metric for speech audibility. Unfortunately, longitudinal studies suffer from other disadvantages relating to population size, loss of follow-up etc., and their duration can approach the limits of the clinician’s working life span. The special characteristics of the study population and methods—neither the WRS_max_ nor hearing-aid scores were measured—differ from the studies mentioned above. This impedes a direct comparison with the above-mentioned studies and therefore does not imply a contradiction amongst them.

In summary, increased PTSL is the most common expression of ARHL. However, there is evidence that a number of other auditory functions are affected as well (Profant et al., 2019). These functions decline with increasing age and the PTSL does not predict speech recognition sufficiently well.

The goal of this study is to describe the relationship between hearing loss, age, and speech recognition by means of a machine-learning algorithm (Random Forest Regression, RFR, Breiman, 2001). RFR is an algorithm that uses an ensemble method of decision-tree-based regressions to determine a response from a set of input variables. It does not rely on any particular assumptions regarding data distribution. This algorithm is applied to a large data set from routine clinical audiometry in order to investigate the influence of age. The result is a representation of the relationship between pure-tone thresholds and age on the input side and speech recognition on the target side. The model reflects the influence of the age-related distortion component on speech perception.

Additionally, the results of the RFR model will be compared with those of a generalised linear model (GLM) approach. In contrast to the RFR, the GLM requires assumptions about the qualitative relation between input and target variables, whereas the RFR does not need a pre-defined equation framework.

In order to categorise pure-tone thresholds, standard audiograms as proposed by Bisgaard et al. (2010) are used as model input. Both derived models (the RFR and GLM) will be applied to these standard audiograms.

## Materials and Methods

Audiometric data were retrieved from a clinical data base at the Audiological Department of Erlangen University Hospital. From the routine audiometric measurements, pure-tone thresholds for both bone and air conduction were extracted. Additionally, speech recognition scores for monosyllabic word lists of 20 items for each presentation level of the Freiburg Test (Hahlbrock, 1957) were evaluated. The complete discrimination function, ranging from 65 dB_SPL_ up to 120 dB_SPL_ was measured. All measurements had been conducted in clinical routine in sound-shielded booths with clinical class A audiometers (AT900/AT1000 AURITEC Medizindiagnostische Systeme GmbH, Hamburg, Germany). Approval for this study was received from the Institutional Review Board of the University of Erlangen (Ref. No. 162_17 Bc). All methods were carried out in accordance with relevant guidelines and regulations.

### Data Preparation

Among 91,991 patients who underwent audiometry at our centre from 2002 to 2020 we identified 53,782 adults aged at least 18 years at the time of first investigation. Initially, the data were screened for repeated measurements. Only the first audiometric assessment of each patient was retained. Subsequently, the data from 107,564 ears (hereinafter “cases”) were checked for a complete set of air and bone conduction thresholds. After removal of incomplete data sets there remained 107,010 cases. In the next step, cases with missing or incomplete speech audiometry data were deleted, whereafter 26,324 cases remained. The data were then screened for cases of mixed hearing loss; the latter was defined as a difference between air and bone conduction thresholds greater than 10 dB for frequencies within the range 0.5–3 kHz. After removal of mixed-hearing-loss cases, the remaining 19,929 cases were checked for inconsistent results (<1%) caused e.g., by simulation or lack of collaboration on the part of the patient. If, within the discrimination function for monosyllabic words, a score larger than zero was observed while the presentation level was below the hearing threshold, the data set for that case was not used. For some cases it was observed that the measurement of the discrimination function had not been fully completed, so that a score of 100% was not reached, with the presentation level well (>15 dB) below the discomfort level. Those cases were removed as well. The 19,801 cases (19,801 ears of 12,040 patients) finally remaining were used for model-building and for error analysis.

The following data were used for analysis:

1. Air-conduction hearing thresholds at 0.125, 0.25, 0.5, 0.75, 1, 1.5, 2, 3, 4, 6, and 8 kHz,
2. Word recognition score at 65 dB_SPL_ (WRS_65_),
3. Maximum word recognition score (WRS_max_) and corresponding level (L_max_).

WRS_65_ describes speech perception at a typical conversational level. While WRS_65_ is primarily dependent on the attenuation and reflects the loss of speech perception ability in everyday life, WRS_max_ describes the maximum information that can be processed to the auditory system. The difference WRS_max_ – WRS_65_ can be used to estimate the acceptance of acoustic amplification (Halpin and Rauch, 2012).

In order to summarise audiometric constellation of our study population we used an established WHO classification (Olusanya et al., 2019). The average of hearing thresholds, measured at 0.5, 1, 2, and 4 kHz (4FPTA) was used to classify according to the WHO categories: WHO_0_ (≤ 25 dB_HL_), WHO_1_ (26 dB_HL_ < 4FPTA ≤ 40 dB_HL_), WHO_2_ (40 dB_HL_ < 4FPTA ≤ 60 dB_HL_), WHO_3_ (60 dB_HL_ < 4FPTA ≤ 80 dB_HL_) or WHO_4_ (80 dB_HL_ < 4FPTA). The Kruskal–Wallis Test was used for group comparisons of the medians for WRS_65_ and WRS_max_.

### Model Setup

For data analysis, model calculation, statistics and figures, the software Matlab *R2019B* including the *Statistics and Machine Learning Toolbox V11.6* (The Mathworks Inc. Natick, Massachusetts) was used. Data were rounded before the RFR model calculation: hearing thresholds to 5 dB and the patients’ ages to life decades. Two models (GLM and RFR) were used to describe the relationship between age and PTSL as input variables and speech recognition variables (WRS_65_, WRS_max_ and L_max_) as target variables. Equation 1 describes the applied GLM for the target variables WRS_65_ and WRS_max_. Equation 2 describes the GLM for L_max_.:


(1) $$WRS\left[\%\right]=\frac{100}{1+e^{-\left(\beta_{0}+\sum_{i=1}^{i=11}\beta_{i}\cdot PTSL_{i}+\beta_{12}\cdot Age\right)}}$$

(2) $$L_{max}\left[dB\right]=\beta_{0}+\sum_{i=1}^{i=11}\beta_{i}\cdot PTSL_{i}+\beta_{12}\cdot Age$$

PTSL_i_ refer to the air-conduction hearing thresholds at the test frequencies 125 Hz to 8 kHz as mentioned above. In order to represent correctly the overall data distribution according to age and 4FPTA, a stratified fivefold cross-validation was applied. In detail, both models, the RFR and GLM, were trained with 80% of the data (training group). The models were then tested in the remaining 20% of the study population (test group). Before group assignment, the data sets were sorted according to 4FPTA and age. Subsequently, every fifth data set was assigned to the test group. This procedure was repeated five times with disjoint training and test sets. The pure-tone thresholds at all frequencies and the patients’ age were input variables, while the WRS_65_, WRS_max_ and L_max_ were targets. For each of the three output variables a separate model was built.

As a parameter for optimisation and estimating the RFR performance, the median absolute error (MAE, resulting from measured minus predicted score) was used as cost function for both the training group and the test group. The MAE of the test group varied up to 25% for different parameters.

For a large range (50–1,000) of the number of learning cycles (equivalent to number of decision trees) the resulting MAE varied by less than 10%. Finally, a value of 100 for the number of learning cycles was used. A small effect on the MAE was found for the other parameters as well. In summary, the following values were used for the Matlab function “fitrensemble()”: “MergeLeaves” = off, the decision tree does not merge leaves. “MinLeafSize” = 5, the minimum number of observations per leaf. “MinParentSize” = 10, the minimum number of observations per branch node. “NumVariablesToSample” = square root of the number of predictors for classification. “PredictorSelection” = allsplits, selects the split predictor that maximises the split-criterion gain over all possible splits of all predictors. The number of nodes per binary decision tree, one result of the model calculation, varied for each model: around 2,150 for WRS_max_, around 2,700 for WRS_65_, and around 3,650 for L_max_.

**FIGURE 1:**
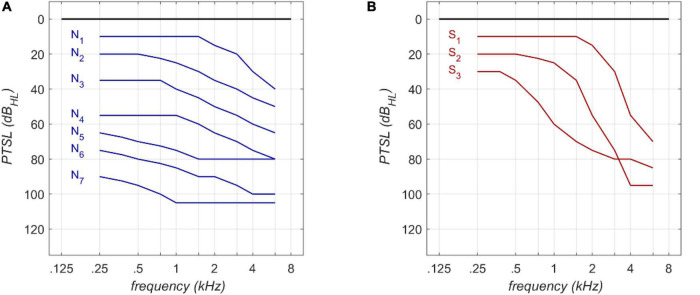
Audiogram types according to Bisgaard et al. (2010) for flat **(A)** and steep **(B)** audiograms.

The RFR and GLM were applied to Bisgaard standard audiograms. These standard audiograms are well established and widely used for audiological investigations (e.g., Tu et al., 2021; van Beurden et al., 2021). They are based on a large clinical data base. The standard set comprises ten standard audiograms (see Figure 1) covering a frequency range of 250 Hz to 6,000 Hz. Flat and moderately sloping (N_1_–N_7_) and steep (S_1_–S_3_) audiograms are considered. Higher indices correspond to greater PTSL.

## Results

**FIGURE 2:**
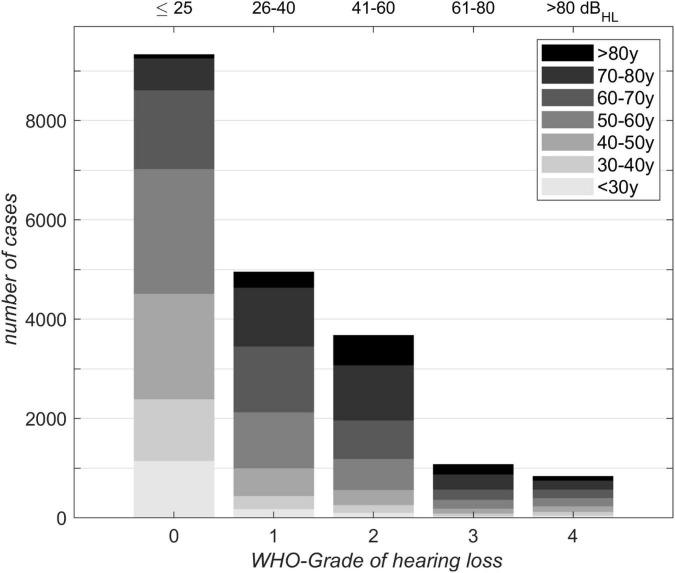
Distribution of 19,801 cases with respect to age for different WHO grades of hearing loss. Corresponding 4FPTA ranges are shown on the upper x-axis.

**FIGURE 3:**
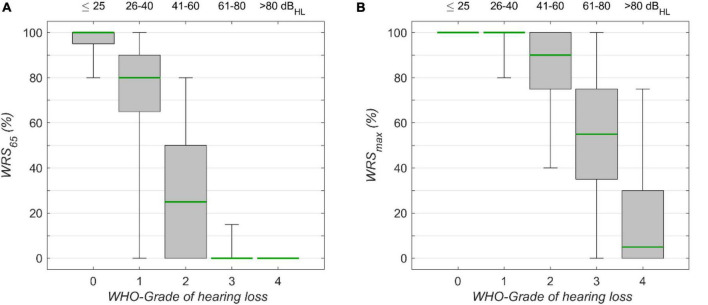
The monosyllabic score **(A)** at 65 dB_SPL_ presentation level (WRS_65_) and **(B)** the maximum word recognition score (WRS_max_), for different WHO grades of hearing loss. Corresponding 4FPTA ranges are shown on the upper x-axis. The boxplots show medians (green) with 1st and 3rd quartiles. The whiskers denote the 2.5 and 97.5 percentiles.

Figures 2, 3 depict the basic characteristics of the clinical population investigated. The stacked bar plot (Figure 2) shows the case distribution in our clinical population (*N* = 19,801). The mean ages of the different groups were 50, 61, 66, 65, and 59 years for WHO_0_, WHO_1_, WHO_2_, WHO_3_ and WHO_4_. The vast majority (77%) of cases involved persons between 40 and 80 years of age. The subjects aged 40–80 years dominated all WHO grades except WHO_0_. The smallest data coverage with respect to age and hearing loss was observed for very young adults in the WHO_4_ group and for subjects above 80 years of age in the WHO_0_ group.

The speech audiometric results for the model’s target scores, WRS_65_ and WRS_max_, are shown in Figures 3A,B, respectively. For both measures the median decreased with increasing degrees of hearing loss. The Kruskal–Wallis Test yielded significant group effects for WRS_65_ (χ^2^ = 15.055, *p* < 10^–15^, df = 4) WRS_65_ and WRS_max_ (χ^2^ = 11.873, *p* < 10^–15^, df = 4). The interquartile ranges for WRS_65_ were 5, 25, 50, 0, and 0% for WHO_0_, WHO_1_, WHO_2_, WHO_3_, and WHO_4_, respectively. The interquartile ranges for WRS_max_ were 0, 0, 25, 40, and 30% for the corresponding WHO groups. The variability for WRS_65_ was largest for WHO_1_, while for WRS_max_ the largest variability was found for WHO_3_. In this rather rough classification the interpretation of some outliers may benefit from additional information about the specific configuration of hearing loss. In particular, the WHO classification employs the hearing thresholds at only four frequencies, while other frequencies are not considered. The lowest quartile of the WHO_0_ cases shows a WRS_65_ lower than 95%. In this subgroup the mean threshold for high frequencies (>4 kHz) was 48 dB_HL_, while for the cases with WRS_65_ above 95% in the WHO_0_ group the mean threshold for high frequencies was 25 dB_HL_ in the WHO_0_ group.

### GLM and RFR

Tables 1–3 show the derived GLM parameters β for each target variable including statistical parameters. For the word recognition scores, WRS_65_ and WRS_max_, the lowest frequency (125 Hz) did not contribute significantly to the model output. None of the other frequencies provided a consistent picture. For L_max_ all but one frequency (750 Hz) contributed significantly to the target variable. For the subject’s age the GLM revealed a significant effect on all target variables. For comparison, the permutation feature importance of the RFR is added in the right-hand column of Tables 1–3. Larger values for a feature indicate a greater impact on the target variable.

**TABLE 1:** GLM parameters for target variable WRS_65_.

Input variable, corresponding measure	GLM statistics for target WRS_65_				RFR-WRS_65_ permutation feature importance
	Estimate	Standard error	t-statistic	*p*	
β_0_, constant	6.5568	0.0308	212.44	0	n. a.
β_1_, PTSL_125 Hz_	0.0013	0.0013	0.95	0.34	1.3
β_2_, PTSL_250 Hz_	–0.0269	0.0018	–15.04	<0.001	1.9
β_3_, PTSL_500 Hz_	–0.0188	0.0017	–11.20	<0.001	1.9
β_4_, PTSL_750 Hz_	–0.0071	0.0019	–3.80	0.00015	0.97
β_5_, PTSL_1000 Hz_	–0.0169	0.0015	10.99	<0.001	2.0
β_6_, PTSL_1500 Hz_	–0.0190	0.0012	–15.30	<0.001	0.97
β_7_, PTSL_2000 Hz_	–0.0212	0.0011	–19.02	<0.001	1.5
β_8_, PTSL_3000 Hz_	–0.0133	0.0010	–13.11	<0.001	1.6
β_9_, PTSL_4000 Hz_	–0.0100	0.0009	–10.60	<0.001	1.7
β_10_, PTSL_6000 Hz_	–0.0152	0.0008	–20.07	<0.001	2.0
β_11_, PTSL_8000 Hz_	0.0002	0.0005	0.48	0.63	1.3
β_12_, Age	–0.0122	0.0005	–26.95	<0.001	2.0
	312,280 observations, 312,267 error degrees of freedom χ^2^-statistic vs. constant model: 2.10^5^, *p*-value < 0.0001				

*For comparison the permutation feature importance of the RFR was added in the right column.*

**TABLE 2:** GLM parameters for target variable WRS_max_.

Input variable, corresponding measure	GLM statistics for target WRS_max_				RFR-WRS_max_ permutation feature importance
	Estimate	Standard error	t-statistic	*p*	
β_0_, constant	7.1589	0.0425	168.41	0	n. a.
β_1_, PTSL_125 Hz_	0.0011	0.0007	1.44	0.15	0.76
β_2_, PTSL_250 Hz_	–0.0047	0.0014	–3.36	0.00079	1.1
β_3_, PTSL_500 Hz_	–0.0135	0.0019	–7.08	<0.001	1.5
β_4_, PTSL_750 Hz_	–0.0032	0.0024	–1.30	0.19	1.2
β_5_, PTSL_1000 Hz_	–0.0136	0.0021	–6.41	<0.001	0.81
β_6_, PTSL_1500 Hz_	–0.0168	0.0018	–9.13	<0.001	1.2
β_7_, PTSL_2000 Hz_	–0.0142	0.0017	–8.38	<0.001	0.81
β_8_, PTSL_3000 Hz_	–0.0081	0.0015	–5.42	<0.001	1.1
β_9_, PTSL_4000 Hz_	–0.0008	0.0013	–0.63	0.53	1.0
β_10_, PTSL_6000 Hz_	–0.0132	0.0009	–14.22	<0.001	2.1
β_11_, PTSL_8000 Hz_	0.0012	0.0005	2.30	0.022	1.3
β_12_, Age	–0.0152	0.0005	–27.81	<0.001	1.4
	317,840 observations, 317,827 error degrees of freedom χ^2^-statistic vs. constant model: 9.10^4^, *p*-value < 0.0001				

*For comparison the permutation feature importance of the RFR was added in the right column.*

**TABLE 3:** GLM parameters for target variable L_max_.

Input variable, corresponding measure	GLM statistics for target L_max_				RFR- L_max_ permutation feature importance
	Estimate	Standard error	t-statistic	*p*	
β_0_, constant	48.7010	0.2552	190.81	0	n. a.
β_1_, PTSL_125 Hz_	–0.0505	0.0093	–5.42	<0.001	1.3
β_2_, PTSL_250 Hz_	0.0582	0.0160	3.64	0.00028	1.5
β_3_, PTSL_500 Hz_	0.0908	0.0195	4.66	<0.001	1.4
β_4_, PTSL_750 Hz_	–0.0016	0.0227	–0.07	0.94	1.4
β_5_, PTSL_1000 Hz_	0.0437	0.0194	2.25	0.024	0.90
β_6_, PTSL_1500 Hz_	0.0719	0.0162	4.45	<0.001	1.4
β_7_, PTSL_2000 Hz_	0.0894	0.0143	6.25	<0.001	1.5
β_8_, PTSL_3000 Hz_	0.1012	0.0119	8.48	<0.001	2.6
β_9_, PTSL_4000 Hz_	0.0610	0.0104	5.84	<0.001	2.1
β_10_, PTSL_6000 Hz_	0.0627	0.0088	7.15	<0.001	1.9
β_11_, PTSL_8000 Hz_	0.0407	0.0059	6.92	<0.001	2.4
β_12_, Age	0.1617	0.0050	32.38	<0.001	2.6
	15,892 observations, 15,879 error degrees of freedom F-statistic vs. constant model: 4.10^3^, *p*-value < 0.0001				

*For comparison the permutation feature importance of the RFR was added in the right column.*

Table 4 summarises the performance of the model as assessed by MAE for both the training and the test group by means of fivefold cross-validation. The results are given separately for the GLM and the RFR model. Owing to the composition of our study population the WHO_0_ is by far the largest group. The MAE of this group would have dominated the overall summary. For this reason, Table 4 shows the error estimation for each grade of hearing loss separately. Evidently, there was a great variation of the MAE among the WHO groups. With the RFR the largest errors were observed in WHO_2_ for the WRS_65_ group and in WHO_3_ and WHO_4_ for WRS_max_. For those WHO groups the MAE of the training and test groups differed by a factor of 1.5 to 1.7. Unlike the RFR, the GLM yielded comparable MAE for the training and test groups.

**TABLE 4:** Median absolute error and its standard error of the RFR and GLM model for the monosyllabic score at a presentation level of 65 dB_SPL_ (WRS_65_), the maximum word recognition score (WRS_max_) and the presentation level for the maximum word recognition score, L_max_.

Target	Cost function	Subgroup		WHO_0_	WHO_1_	WHO_2_	WHO_3_	WHO_4_
WRS_65_	MAE (percentage points)	Training	RFR GLM	1.03 ± 0.04 1.91 ± 0.01	5.60 ± 0.06 9.04 ± 0.04	8.01 ± 0.11 14.36 ± 0.17	0.06 ± 0.02 2.09 ± 0.04	0.004 ± 0.002 0.018 ± 0.001
		Test	RFR GLM	1.53 ± 0.08 1.91 ± 0.01	8.88 ± 0.24 9.04 ± 0.24	12.70 ± 0.59 14.42 ± 0.79	0.10 ± 0.05 2.10 ± 0.11	0.004 ± 0.002 0.018 ± 0.001
WRS_max_		Training	RFR GLM	0.04 ± 0.01 0.57 ± 0.01	1.54 ± 0.02 3.04 ± 0.03	5.65 ± 0.08 8.71 ± 0.08	10.79 ± 0.36 18.92 ± 0.41	6.76 ± 0.27 8.06 ± 019
		Test	RFR GLM	0.06 ± 0.01 0.57 ± 0.01	2.27 ± 0.05 3.04 ± 0.07	9.06 ± 0.25 8.71 ± 0.26	17.24 ± 1.94 18.91 ± 1.51	11.28 ± 1.56 7.82 ± 0.78
L_max_	MAE (dB)	Training	RFR GLM	3.44 ± 0.04 5.31 ± 0.04	3.26 ± 0.05 5.30 ± 0.08	3.39 ± 0.06 5.85 ± 0.06	3.14 ± 0.13 5.81 ± 0.11	2.75 ± 0.16 8.63 ± 0.25
		Test	RFR GLM	5.09 ± 0.17 5.31 ± 0.18	5.26 ± 0.15 5.34 ± 0.23	5.41 ± 0.23 5.85 ± 0.30	5.16 ± 0.46 5.85 ± 0.55	4.42 ± 0.46 8.65 ± 1.15

### Application of the Model

**FIGURE 4:**
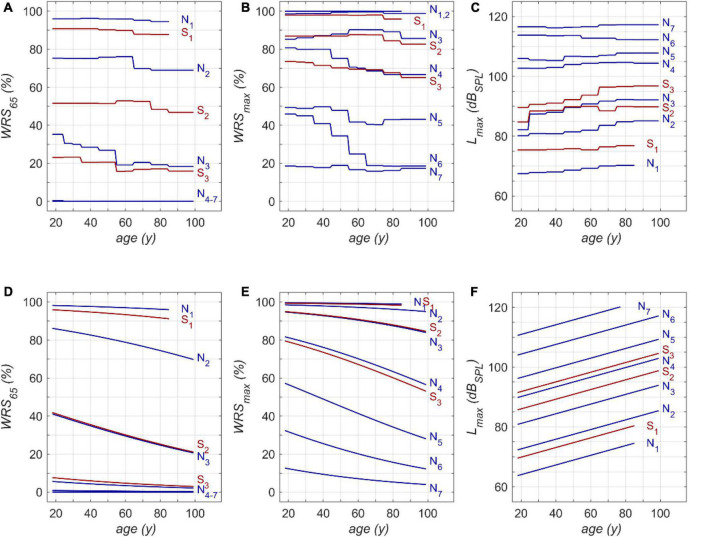
Model results for RFR **(A–C)** and GLM **(D–F)**: **(A)** age dependence of monosyllabic score at 65 dB_SPL_ presentation level (WRS_65_), **(B)** maximum word recognition score (WRS_max_) and **(C)** applied presentation level (L_max_). N_1_ to N_7_ and S_1_ to S_3_ refer to the Bisgaard types of audiograms. The second row **(D–F)** shows the results of the GLM equivalent to the upper row **(A–C)**. WRS_65_ and WRS_max_ were calculated according to equation 1 **(D,E)**. L_max_ was calculated according to equation 2 **(F)**.

One possible application of the model is shown in Figure 4. The model input was one of the standard audiograms (N_1_–N_7_, S_1_–S_3_) and the subjects’ age was varied between 18 and 99 years. Owing to the relation between age and hearing thresholds hardly any subjects were in our population aged > 85 years for N_1_ and S_1_. Therefore, this range was excluded from model calculations.

Figures 4A,D show that both models indicate a decrease in WRS_65_ with increasing age of up to 20 percentage points across the whole life span. The GLM suggests a rather constant decline of speech recognition over life span. The RFR on the other hand yields specific periods with different amounts of age-dependent decline. The largest decrease was observed for N_3_ in the fifth life decade with 10 percentage points per decade.

The RFR results become even more complex if the WRS_max_ and L_max_ are considered, as shown in Figures 4B,C, respectively. The presentation level shows, for all types except N_6_, an increased presentation level for WRS_max_ with increasing age. A considerable decrease in score can be observed in N_6_, accompanied by a slight but significant decrease of L_max_. For the N_4_ and S_3_ types the RFR model gives a significant decrease in WRS_max_ which is somehow weakened by an increased presentation L_max_ for this type. For all other types the WRS_max_ does not change with age. However, for these types the RFR model results in an increased presentation level. In comparison, the GLM output indicates a decline for WRS_max_ over age while L_max_ increases for all audiogram types. For both models a decrease of up to 25 percentage points across the whole life span was observed.

## Discussion

The analysis of a large clinical database allows the description of the age-related decline of speech perception in detail. In comparison with previous studies, more detailed information about the time course and amount of degradation was achieved by means of RFR. Both models, the GLM and the RFR, describe an age-related decline in speech recognition after being corrected for PTSL. The GLM is based on predefined hypotheses and confirms significant age effects. Inevitably, the relationship between age and speech scores follows the underlying functional relations. The GLM results in an age-related decline for WRS_max_ of about 3–4% per decade for N_4_-N_6_, and S_3_. For all other audiogram types smaller effects were found owing to saturation effects. This is in concordance with previous studies (Jerger, 1973; Dubno et al., 2008). WRS_65_ decreases at a rate of up to 2.5% per decade for mild hearing losses, i.e., N_2/3_ and S_2_. For the other audiogram types the GLM yielded smaller rates of decline. Owing to the lower presentation level of 65 dB_SPL_ floor effects were observed even for moderate hearing losses, i.e., N_4–7_. The RFR model yielded more specific information about the time course and rate of decline. Additionally, the RFR model allows the quantitative description of the two basic effects of hearing loss and its relation to age: On the one hand the impact of the attenuation component of ARHL, and on the other hand the impact of the distortion component of ARHL. This could be achieved by keeping constant the model input variables representing PTSL (attenuation), and by modifying the model input variable representing age. It therefore offers the opportunity to overcome a bias that was immanent in previous investigations (Jerger, 1973; Marshall and Bacon, 1981; Dubno et al., 1997; Hoppe et al., 2014; Müller et al., 2016) by isolating age-related hearing threshold elevation from age-related decline in speech recognition as such.

This study should not be misunderstood as an attempt to predict speech recognition scores on the basis of PTSL. These scores have to be measured individually. The large variability of individual scores necessitates speech audiometry. The purpose of the model in this study was to analyse the impact of age for larger patient populations with respect to specific audiogram types. It can be seen in Figure 4 that those age-related changes are present for the entire duration of adulthood. However, apart from the fact that higher age relates to lower speech recognition scores, no common quantitative trend, for any age groups or PTSL, can be discerned. This may be regarded as the major outcome of the RFR model calculations. The measurable age-related decline in speech recognition depends on the age range considered, the specific audiogram, and the specific application of speech audiometry. Owing to saturation effects of the WRS_65_ measured at typical conversation level, we observed the largest age effect for moderate hearing losses (N_3_-type audiograms). For the WRS_max_ measured at substantially higher levels, the largest effects were observed for audiogram types corresponding to severe hearing losses (N_4_, N_5_, N_6_). This result of the RFR is in agreement with findings of Jerger (1973). Even though the variability in his data is considerable (as in our data) one may conclude that a stronger age-related decline can be observed for later life decades and greater hearing loss. Additionally, Jerger’s data also indicated that the onset of age-related decline may occur already at younger age. This is in line with our results where the RFR model e.g., yielded for N_6_ the strongest decline for WRS_max_ of 20% per decade around the fifth and sixth life decade.

According to the RFR, the decrease in the WRS_max_ was counterbalanced by an increased presentation level for all audiogram types except N_6_. The N_6_ -type audiogram showed the largest age-related decline in speech recognition. The decreased tolerance of higher presentation levels may have contributed to this decline. This might reflect certain underlying pathomechanisms that are more likely to be present in patients with this audiogram type compared with others. Complementary to attenuation and distortion, a causal and more differentiated breakdown with respect to presbyacusis was proposed early on. Finally, five main types were proposed, namely sensory, neural, metabolic, mechanical, and vascular presbyacusis (Schuknecht, 1964; Johnsson and Hawkins, 1972). This was complemented by the term central presbyacusis in order to reserve the term neural for degeneration of the cochlear nerve. Sensory presbyacusis is congruent with the attenuation component and is, as pointed out above, represented by the audiogram type as a fixed parameter in Figure 4. The effects of all the other types of presbyacusis are included in the specific relationships between age and WRSs, respectively, L_max_. Moreover, the specific and different root causes may potentially explain why, for some degrees of hearing loss, different changes in speech perception occur in different life decades. However, possible interactions between—or even independent mechanisms—of the main types of presbyacusis are still not completely understood (Bao and Ohlemiller, 2010; Profant et al., 2019).

It is not possible to confirm all these explanatory hypotheses by retrospective data analyses, a fact that clearly underlines the limits of our study design. We found differences in age effects in comparison with some of the studies referred to above. This is partly due to the neglect of hearing loss at higher frequencies for the elderly in those studies. On the other hand, for some hearing losses and audiogram types, this study may underestimate age effects, as ceiling effects of speech tests in quiet are included. Another aspect of this study is the inclusion of a considerable number of subjects with mild hearing loss, as seen in group S_1_. Even in that group, age effects play a part. Especially the WRS_65_ illustrates how everyday communicative ability in quiet might be already affected by mild to moderate hearing loss in a population in which the use of hearing aids does not reach the penetration level needed (Halpin and Rauch, 2012).

Other possible applications of the RFR model are related to acoustic amplification with hearing aids: As shown in Figure 4, in all groups except N_6_, the level for best speech recognition (L_max_) increases with age at about 0.5 dB per decade. This may indicate that older people may benefit from larger sound pressure levels for speech recognition, i.e., greater amplification, when provided with a hearing aid. As far as we know, current amplification strategies do not take this into account. On the other hand, one has to consider that in some pathologies more amplification might be detrimental rather than beneficial (Halpin and Rauch, 2012).

The age dependence of the WRS_max_ found in our study may be used to improve studies evaluating the outcome of hearing aid use: The WRS_max_ or an equivalent measure is often used as reference for the measurement of successful hearing aid provision or other acoustic amplification (Halpin and Rauch, 2012; Hoppe et al., 2014; Müller A. et al., 2017; Maier et al., 2018a,b), for investigation of age-related changes in cognition (Kronlachner et al., 2018), and for speech-perception-related studies in general (Müller J. et al., 2017). A consideration of both age and specific audiogram type could potentially decrease the variability of results. Furthermore, the functional relation between audiogram types and speech perception as presented here can be used to link epidemiological studies on hearing loss (Sohn and Jörgenshaus, 2001; von Gablenz et al., 2017, 2020; Chang et al., 2019; Löhler et al., 2019; Cantuaria et al., 2021) with speech recognition.

### Comparison of the Two Model Approaches

The need for pre-defined hypotheses may be considered a weakness of the GLM, as all model results inevitably follow the underlying analytical equations. If an effect for certain audiogram types is found, the GLM yields a smooth decline over all life decades. The RFR is able to take varying rates of decline in different life decades into account if variation indeed takes place in the study population. Overall, as shown in Table 4, for most of the WHO groups the RFR yielded smaller MAE for the test groups compared with the MAE yielded by the GLM. However, the differences obtained between MAE in the training and test groups by RFR indicate some degree of overfitting. This was not the case for the GLM.

The impact of audiometric test frequencies on the calculated WRS is different for the two model approaches. The GLM is less suitable to reflect the impact of low and high frequency hearing loss for all WHO groups. In cases with mild hearing loss higher frequencies have a greater impact: Typically, the low frequencies show low variability and fail to explain the variability in the scores. Vice versa, for cases with severe hearing loss the PTSL for high frequencies are already near or at the audiometer limits. Consequently, the GLM explains the variability in the scores by utilising PTSL in the low–frequency range. As a result for all WHO groups, the GLM suggests that there is no effect of the highest and lowest test frequencies (Tables 1, 2). Some other findings, such as the absence of an effect at 750 Hz on the WRS_max_ in Table 2, can be considered as typical signs of an overdetermined system. The measurement at 750 Hz does not provide any additional information compared with the adjacent frequencies and vice versa. *A priori*, there is no audiological rationale for removing single test frequencies.

### Limitations of the Study

An important limitation of this study is the restriction to a specific language and test. However, with respect to other languages and speech material the comparison of recent studies (Holden et al., 2013; Hoppe et al., 2019) suggests that the test we used is comparable to the English Consonant-Vowel-Nucleus-Consonant (CNC) test (Causey et al., 1984).

Secondly, the outdated but established calibration procedure for the Freiburg monosyllable test at 65 dB_SPL_ (Holube et al., 2019) is roughly comparable to a level of 60 dB_A_. Consequently, L_max_ should be corrected by about 5 dB for a comparison e.g., with CNC results.

The disadvantage of binary decision trees is the high chance of overfitting. The use of a random-forest method decreases this risk. However, a factor of up to 1.7 between the MAEs in the test group as compared with the training group still indicates some degree of overfitting. Even the considerable size of the study population and the clustering of input variables do not entirely prevent this risk. Additionally, there are some intrinsic sources of unexplained variability. Even after thorough data–cleaning as described above, the population may still have included mild cases of aggravation, simulation or dissimulation. There was also a small number of cases with retrocochlear lesions. This number can be estimated as less than 0.5% in our population by comparison with our patient files and the reported incidence (Lin et al., 2005). The unilateral processing of the data without the contralateral status as additional input variable is a potential shortcoming and should be therefore subject to future studies as well.

An RFR model inevitably reflects the characteristics of the clinical population that contributed to the training. The group characteristics differ from those of their peers outside a clinic. Finally, the model reflects the statistical characteristics of a population, and not causal relationships.

## Conclusion

A random-forest regression model allowed the estimation of age-related decline of speech recognition in quiet, completely separated from the effect of pure-tone sensitivity loss. Noticeable declines were found across the whole duration of adulthood and for all audiogram types. Model calculations resulted in a decrease of up to 25 percentage points word recognition scores across the whole life span. Depending on the specific hearing loss, the RFR model indicated a maximum decline of up to 10 percentage points in certain life decades. The decline can be attributed to an increased distortion component related to presbyacusis which is not represented by pure-tone audiometry. The careful derivation of working hypotheses from our data has the potential to provide greater insight into the relationships between pure-tone sensitivity loss, specific audiogram types and age.

## Data Availability Statement

The raw data supporting the conclusions of this article will be made available by the authors, without undue reservation.

## Ethics Statement

The studies involving human participants were reviewed and approved by Ethik-Kommission, Friedrich-Alexander-Universität Erlangen-Nürnberg (FAU). The patients/participants provided their written informed consent to participate in this study.

## Author Contributions

UH: conceptualization. UH and TH: formal analysis, writing original draft, methodology, software, validation, and visualization. UH and HI: investigation, project administration, and resources. UH, TH, and HI: writing – review and editing. All authors contributed to the article and approved the submitted version.

## Conflict of Interest

TH was employed by Cochlear Deutschland GmbH & Co. KG. The remaining authors declare that the research was conducted in the absence of any commercial or financial relationships that could be construed as a potential conflict of interest.

## Publisher’s Note

All claims expressed in this article are solely those of the authors and do not necessarily represent those of their affiliated organizations, or those of the publisher, the editors and the reviewers. Any product that may be evaluated in this article, or claim that may be made by its manufacturer, is not guaranteed or endorsed by the publisher.
